# How African Americans With Severe Mental Illness and Trauma Experience Diet and Exercise

**DOI:** 10.52678/001c.74364

**Published:** 2023-04-14

**Authors:** Lindsay Sheehan, Janis Sayer, Mariyam Siddiqi, Sang Qin, LaToya Glover

**Affiliations:** 1 Illinois Institute of Technology; 2 Advocates for Human Potential (United States)

**Keywords:** African American, serious mental illness, trauma, diet, exercise, obesity

## Abstract

African Americans have a higher prevalence of obesity and obesity-related diseases than other racial/ethnic groups; among persons with serious mental illness (SMI), African Americans fare worse as well. This qualitative study focused on the perceptions of African Americans with SMI in regard to 1) their experiences with diet and exercise behaviors post trauma and 2) how diet and exercise programming can address trauma. A community-based participatory research (CBPR) team developed the interview guide, research protocols, and conducted three focus groups. The team used thematic analysis to analyze the data. Participant (*N* = 27) responses on the experience of trauma were coded into the following themes: 1) emotional eating, 2) appetite loss, 3) hesitancy to exercise due to community violence, 4) staying home due to mental health symptoms, and 5) substance use. Themes around how programming can address trauma included: 1) support, 2) communication, 3) strategies to avoid trauma, and 4) engagement in programming. Findings suggest the need for human service professionals to infuse trauma-informed communications and practices throughout programming, incorporate peer-led services and address concerns related to community violence.

African Americans with serious mental illness (SMI) experience high obesity rates, resulting in severe medical morbidity and reduced quality of life (Cook et al., 2016). One dimension of social determinants of health—neighborhood and environment—contribute to this disparity (Robins et al., 2022). African Americans from underserved and disenfranchised communities are exposed to trauma and neighborhood violence that impact their health and impede access to health and human services (Frazer et al., 2018). These disparities demand that trauma-attuned practices be implemented by human services professionals (Mihelicova et al., 2018; U.S. Department of Health and Human Services, 2014). This qualitative thematic analysis explores the phenomenological experiences of African Americans with SMI with goals of highlighting their perspectives on: 1) how trauma influences their diet and exercise and 2) how diet and exercise programming can be sensitive to experiences of trauma.

People with SMI are disproportionately burdened by physical illnesses, including diabetes, cardiovascular disease, hypertension, stroke, metabolic syndrome and obesity-related cancer, which occur at higher rates than in persons without SMI and contribute to shortened lives (Janssen et al., 2015). While the causes of these health disparities are multiple and complex, modifiable risk factors contribute to chronic disease, including obesity, which affects up to 55% of people with SMI (Janssen et al., 2015). Similarly, 38% of African American men and 56% of African American are categorized as obese (National Center for Health Statistics, 2019). Among persons with SMI, African Americans are at a greater risk for obesity than other groups (Carliner et al., 2014).

Human services professionals have been increasingly concerned with reducing health disparities (Fichtenberg et al., 2020) and have developed wellness interventions around healthy eating and exercise (Speyer et al., 2019). However, these wellness interventions have shown limited efficacy in improving outcomes for individuals with SMI. In a meta-analysis of 41 randomized controlled trials of weight management programs for SMI, Speyer and colleagues (2019) concluded that weight loss across interventions was statistically significant but too small in magnitude to be clinically relevant. Rocks and colleagues (2022) conducted a meta-analysis of 25 studies focusing just on diet and nutrition interventions in SMI, finding similar low effect sizes; only 8 out of 17 interventions resulted in statistically significant reductions in weight. Given these limitations with current interventions, approaches that are culturally sensitive and trauma-informed, may be a next step in approaching this seemingly intractable issue.

Social determinants of health such as economic stability, education, neighborhood conditions, and health care access and quality, contribute to health disparities for African Americans with SMI (Allen et al., 2014; Robins et al., 2022). For example, low income and minority neighborhoods are often characterized by a scarcity of healthful, affordable food choices, an abundance of fast-food restaurants, long distances to supermarkets (Bower et al., 2014) and lack safe areas to exercise (Lane & Davis, 2022). Individuals with SMI have unique barriers to engaging in wellness programs, which include heightened stress responses, depression-related anhedonia, and lack of support (Firth et al., 2016). For example, over 60% of individuals with SMI reported that low mood and stress interfered with exercise (Firth et al., 2016). Similarly, African Americans with SMI have unique concerns around diet and exercise that should be taken into account when developing interventions (Sayer et al., 2019).

## Trauma and Its Relationship to Obesity

Trauma is another contributor to obesity that disparately impacts African Americans with SMI. Some individuals who are exposed to a traumatic event will develop posttraumatic stress disorder (PTSD), a psychiatric condition marked by chronic hyperarousal and fear, which may drive mechanisms leading to weight gain (Kubzansky et al., 2014). In observational studies, persons with PTSD are more likely, relative to controls, to be obese (Bartoli et al., 2015). However, very few prospective studies have examined the link between obesity and PTSD/PTSD symptoms (Kubzansky et al., 2014). In a longitudinal study with over 50,000 participants that examined obesity risk following trauma exposure in women, researchers found that the presence of even one symptom of PTSD increases the risk of obesity (Kubzansky et al., 2014). Controlling for depression, women exposed to trauma who endorsed at least one symptom of PTSD had a significantly increased risk of obesity as compared with those without trauma exposure. Likewise, women who had experienced trauma but did not experience any PTSD symptoms did not have a higher risk of obesity than women without trauma exposure. Greater numbers of PTSD symptoms were related to greater BMI increases over time (Kubzansky et al., 2014). In a U.S. nationally representative sample, African Americans were more likely than other racial or ethnic groups to experience PTSD (Alegría et al., 2013). As a marginalized population, African Americans experience cumulative traumas, such as racial discrimination, historical trauma, poverty, and adverse childhood experiences (Myers et al., 2015). Likewise, individuals with SMI are more likely than the general population to be diagnosed with PTSD (Mueser et al., 2004), suggesting the need for greater attention to the link between trauma and obesity in this intersectional population.

One distinctive type of trauma—community violence— has a disparate impact on African Americans (Hampton-Anderson et al., 2021) and is associated with excess weight gain. This link may be attributed to the fact that individuals in violence-plagued neighborhoods struggle finding healthy food sources (Miller et al., 2021) and accessing safe areas to exercise (Brown et al., 2014; Wright et al., 2017). Living in an unsafe community exposes people to chronic psychological stress that is frequently related to eating and metabolic problems, for example, emotional eating, insulin resistance, and craving for hyperpalatable foods (Yau & Potenza, 2013). The anticipation of violent incidents may dissuade individuals from outdoor activities and cultivate a homebound lifestyle (Burdette & Hill, 2008; Stolzenberg et al., 2019). Community violence can shape people’s diet and activity habits, resulting in long-term adverse effects.

While previous studies show that trauma may influence diet, physical activity, and body weight, this is underexplored in African Americans with SMI. In a systematic review of qualitative studies on African American adults (who did not have SMI), the authors found numerous individual-level and environmental barriers to physical activity participation, including motivation, physical disability, fatigue, lack of money or limited community resources (Siddiqi et al., 2011). However, trauma was not mentioned as a barrier in any of the reviewed studies.

Given the rising numbers of trauma survivors in the service systems, human service providers have an ethical imperative to recognize the impact of trauma and appropriately respond on the individual, interpersonal, and system levels (U.S. Department of Health and Human Services, 2014). While some exercise programming has begun incorporating practices that are sensitive to participant trauma, including “no-touch policies” and better incorporation of participant preference (Darroch et al., 2020), an enhanced understanding of the trauma experience and its influence on diet and exercise can further support the integration of trauma-informed practice in the programs and agencies that provide services to this population. Hence, the present study attempts to fill the gap in this literature by highlighting the perspectives of African American with SMI.

## Purpose Statement and Research Questions

The current study aims to explore how African Americans with SMI experience health-related behaviors post trauma exposure and how the negative impacts of trauma can be mitigated in health promotion programming. Specifically, two research questions guided the development of the subsequent research activities:

1. How do African Americans with SMI experience diet and physical activity behaviors post traumatic experiences?
2. How can a diet and exercise programming be sensitive to trauma experiences?

We opted for a qualitative research design, as it allowed for a more in-depth analysis of topics where there is limited information and can provide specific suggestions for tailoring interventions to this population.

## Methods

### Sampling

Participants (*N*= 27) were recruited via flyers and word-of-mouth through two partner mental health organizations. Three focus groups were held in a large midwestern city and located nearby the mental health organizations. Participants were screened by phone and were considered eligible if they self-reported: 1) being at least 18 years old, 2) identifying as African American, 3) having a SMI, 4) having challenges with weight and 5) having experience with trauma. SMI was defined for participants as having a “mental illness that makes it difficult for you to achieve your life goals.”

### Procedures

The present study used a community-based participatory research (CBPR) design, in which community stakeholders and academic researchers collaborated as equal partners throughout the research process (Sheehan et al., 2021). A CBPR team, which was already meeting weekly for a larger project to develop and test a weight loss intervention for African Americans with SMI, developed the research protocols and conducted focus groups for this qualitative study. The CBPR team included one African American with lived experience of mental illness who served as team leader, two academic researchers, a healthcare provider, and six additional team members who were African American members with lived experiences of both mental health and weight challenges. The CBPR approach ensured that the research approach was culturally relevant and research questions were valuable to effect change in communities. The team obtained approval from the institutional review board at a private midwestern university and team members were paid $25/hour for their work on the research. Participants were compensated with a $50 gift card for attending the 90-minute group. Up to 10 individuals were scheduled per group and between 8 and 10 attended. Sessions were audio recorded and transcribed. To protect confidentiality, participants were identified only by first names during the group and transcripts were coded with numbers instead of names.

### Data Collection

One academic researcher and one CBPR team member co-led each of the three focus groups. Participants filled out a short paper-and-pencil demographic questionnaire and signed the consent form prior to beginning the group. The focus group facilitators explained the purpose of the study, briefly defining and giving examples of potentially traumatic events that might influence weight loss. The facilitator with lived experience briefly introduced themselves, disclosed their lived experience, and emphasized the significance of the research. Facilitators asked participants the following questions: 1) how has trauma affected your eating habits? 2) how has trauma affected your activity or exercise habits? 3) how has trauma affected your participation in health and wellness programs? and 4) what can we include in a weight loss program that would make people who have experienced trauma participate? Facilitators asked clarifying and follow-up questions, and welcomed all participants to share their perspectives.

### Participants

Of the 25 (out of 27) individuals who filled out the demographic questionnaire, about half (*n* = 13) were female and all reported being African American or African American and another race or ethnicity. Age of participants ranged from 34–67 with an average age of 50.3. Participants were advised during the screening that interview questions focused on the *impact* of trauma, and they would not be asked to share their personal traumas in the group. Participants were reminded of this again at the beginning of the focus group session and were provided with mental health and trauma related resources at the end of the focus group. A licensed psychologist was present in the building during the focus group sessions and available to meet with any participant during or after the session.

### Data Analysis

We used thematic analysis to analyze patterns in the data in relation to the two major themes preidentified in our research questions (Marshall & Rossman, 1999). Our analysis used an emergent strategy to identify the full range of subthemes within each theme. Two researchers (JS and SQ) thoroughly read the transcripts, made independent notes, and developed the preliminary subthemes and codebook. These were then presented to the CBPR team (some of whom had observed the focus groups) and discussed. As a result, additional description was added to the codebook to clarify boundaries between subthemes. Using MAXQDA 12 software and the codebook, two researchers (JS and SQ) independently coded the first transcript, then met to discuss “bytes” (distinct pieces of information) that did not seem to fit into pre-established subthemes. This discussion led to some reorganization of subthemes and subsequent amendments to the codebook. At this point, coders had established a Kappa interrater reliability of .80 and coded the remaining two transcripts independently. Next, they reviewed each other’s coding, making notes on any discrepancies or questions. Finally, the two met to discuss and agree on final coding structure. Final themes and subthemes were reviewed with the CBPR team and discussed in terms of implications for future research and program development.

### Trustworthiness

Here we describe our efforts to achieve trustworthiness in this study. First, we sought credibility through a CBPR approach, including a full partnership between key stakeholders and researchers. The interview guide was informed by lived expertise of African Americans with SMI and weight concerns. Interviews were recorded and transcribed verbatim; interrater reliability was established when researchers recorded the transcripts. Transferability of the findings was attained by selecting a diverse sample; participants were recipients of community mental health agencies and prospective users of trauma-sensitive diet and exercise programs. To assure confirmability, themes and supporting bytes that emerged from the transcripts were submitted and discussed with the lead researcher (LS) and CBPR team. Revisions were incorporated into data analysis when a consensus was reached by the team. Regarding dependability, a systematic method was applied to data analysis. A codebook was first established after reviewing the content of the transcripts. Researchers (JS & SQ) followed an iterative process to identify the common themes using MAXQDA, a qualitative data analysis software.

## Results

Participant perspectives on trauma are organized into two themes that correspond to our research questions: 1) Experience of trauma on diet and exercise and 2) how weight loss programs can address trauma. In examining the experience of trauma on diet and exercise, we included participant responses that described how their trauma experience affected eating or physical activity behaviors. These are reflected in the following subthemes: 1) emotional eating, 2) loss of appetite, 3) hesitancy to exercise, 4) staying home, and 5) substance use. Our second theme was categorized into four subthemes: 1) provide support, 2) communication, 3) teach strategies to avoid trauma, 4) engagement and empowerment. We describe each of these in detail below, but first we discuss traumas as described by participants.

### Types of Trauma

Although we did not ask participants to explicitly share the types of trauma they personally experienced, in the context of discussions, many opted to discuss these traumas and we provide a summary here for context. Some participants described the types of trauma they had experienced, which included specific events (witnessing violent deaths, death of loved one, or robberies) and chronic traumas (child abuse, domestic violence, relational violence, living in unsafe neighborhoods, relationships ending). For example, one participant said “My brother was murdered a couple of years back. That led to some problems. Survival of childhood abuse also. That plays a big part of food, which is the drug of choice for me.” Another said, “When the police cars arrive in the neighborhood sometimes 5–7 deep and continuously, I feel like I’m in a movie. ‘Wait a minute, what is this?’ I feel like I’m in a movie.” Participants also described that living with chronic conditions such as mental illness and substance use disorder, and being homeless was traumatizing: “There’s a lot of people that have mental illness and kind of have adapted to it. They not getting treated, the kids aren’t getting treated.” Another participant spoke about experiencing historical trauma as a member of the African American community (“I got ancestral trauma”).

### Theme 1: Influence of Trauma on Diet and Exercise

In examining the influence of trauma on diet and exercise, we included participant responses that described how their trauma experience affected eating or physical activity behaviors. Subthemes included: 1) emotional eating, 2) loss of appetite, 3) hesitancy to exercise, 4) staying home, and 5) substance use.

#### Subtheme 1: Emotional Eating

Several participants (*N* = 7) described emotional eating as a response to trauma. They spoke about eating as a response to trauma-related depression or as a way of coping with anxiety. One said, “I use food as a coping mechanism when dealing with stress and intrusive thoughts.” Another said “Sometimes I get depressed and all I can do is eat. Some people do drugs and drinks, but I eat sweets and carbs. The dollar store has pot pies for a dollar. When I found that out, that was like discovering the fountain of youth.” A few spoke about difficulty making healthy choices when they were stressed or emotional.

#### Subtheme 2: Loss of Appetite

A few participants (*N* = 3) spoke about losing their appetite in response to trauma. One participant said: “Trauma can make you eat and not eat too.” Another said,” If you witness something violent or have a tragic death in your family. You may not eat. You may be so stressed out or hurt so you decide not to eat or eat very little.” They usually described weight loss as occurring shortly after the traumatic event. While this might have resulted in them losing weight and being perceived positively by others because of the weight loss, it was a reflection of their ill health rather than improvement in health.

#### Subtheme 3: Hesitancy to Exercise Due to Community Violence

Several participants (*N* = 11) expressed concern about exercising outdoors, mostly in response to community violence. One said, “You may want to work out or walk but you know what? You’re afraid, you might think shooting can happen anywhere.” Another said, “The basketball courts in these places are empty because people are scared because the gangbangers are going to come. You can have exercise without a regimen like playing basketball with your kids, but you’re in fear to going to these open-air parks because something might happen to you.” One person described a specific incident that changed their behavior: “There was someone who made me feel uncomfortable, so I stopped walking like that. Now I take the shortest route instead of walking the street. Violence has altered the way I do work out.” Participants recognized the differences between their neighborhoods and those of others: “It looks like a lot of Caucasians they run and stuff. They jog. I live by the university. They jog to places because they are safe.” Another said “In safe neighborhoods, they jogging and walking they dogs at night.” One person also noted that regardless of neighborhood, skin color influenced safety, “The white people trickling in [to the neighborhood] but they ain’t stupid either. They know that police will come save them. For African Americans, they see it every day. The white woman feels safe to jog and power walk but I’m not gonna feel safe.” This fear extended to exercise in indoor spaces such as gyms located in unsafe neighborhoods, that experience vandalism and outdated equipment. One person said that exercising and having a toned body might draw scrutiny from gangs: “I live in a gang infested environment. The better the body, the more jealous. You have the pressure of the competition. They [gangs] might try to hurt you.” These participant responses all demonstrate how perceptions of community violence can interfere with engagement in physical activity for this population.

#### Subtheme 4: Staying Home Due to Mental Health Symptoms

Participants (*N* = 8) spoke about often staying in their home and thus giving up opportunities to pursue physical activity and healthy foods. One person described this as “I stay in the house and hibernate.” Another said “People dying back-to-back... it makes me hibernate. Since I don’t want to harm myself, I eat. I stay in the house for days and don’t come outside. Whatever I got in the house, that’s what I eat.” This behavior was often described as being driven by stress, distress or depression in relation to trauma. Stress and anxiety made it more difficult to leave the house and go into a new environment. Depression made it hard to initiate activities outside the house and maintain active routines.

#### Subtheme 5: Substance Use

A few participants (*N* = 2) said that substance use disorder (resulting from trauma) made it hard for them to exercise and maintain a healthy diet. One said, “I didn’t have no eating habits, it was drinking habits when I lost my mom. They all called me a special child because they didn’t think I’d live. When she passed, I started off drinking beer..... I didn’t eat then, just drank.” Another added, “Drugs can make you depressed. You just don’t eat. When the drugs gone, then you wanna eat.” These comments highlight the difficulty of maintaining regular eating habits and nutritional intake while using substances in response to trauma.

### Theme 2: How Weight Loss Programs Can Address Trauma

As part of the interview guide, we asked participants how a diet and exercise programs can be sensitive to traumatic experiences. We categorized these responses into four themes: 1) provide support, 2) communication, 3) teach strategies to avoid trauma, 4) engagement and empowerment.

#### Subtheme 1: Support

Participants (*N* = 5) spoke about ways that weight loss programs could be more supportive and could recognize that trauma is a common experience. They made suggestions on how a weight loss program might provide phone numbers or referrals to trauma support, or set participants up with a mentor to support them on nutrition and activity goals. One participant suggested having motivational speakers who address trauma and give people hope, emphasizing that hearing from others with similar experiences can be powerful: “[Talking with] people that have gone through the same. You will be surprised to see how it can help.” These responses suggest the importance of supports that are embedded within programming and that anticipate participant needs for a higher level of support.

#### Subtheme 2: Communication

A few participants (N = 4) highlighted the importance of communication within the context of services, and how that could be trauma-informed. This included being non-judgmental and sensitive about weight, body type, religious/political beliefs, race/ethnicity and lifestyle. Participants discussed how service providers should avoid pushing their opinions on participants or verbally coercing them to engage in activities that they are not comfortable with. One summed up this point, “Sometimes you just have to be mindful of what you say to people and how you treat them. You have to be able to know how to address them. He might be going through something. Put it this way, your presentation has to do a lot with how he might experience trauma. It might make the person feel better.” Participant responses on this theme stressed the importance of thoughtful communication styles of program facilitators that create a safe and nonjudgmental environment for those with a history of trauma.

#### Subtheme 3: Teach Strategies to Identify and Avoid Trauma

Participants pointed out that many individuals do not recognize their own trauma and the effect that it has on their life. One said, “A lot of people don’t know trauma. Especially for mental illness, people might be traumatized on a daily basis and may not even know it.” Another said, “I think some people have experienced trauma but I don’t think they can identify it. It’s not cut and dry.” Thus, thoughtful communication can help others become aware of and address their trauma. A few participants (*N* = 3) spoke about teaching people strategies to prevent further trauma such as through exercising at home or finding safer spaces (e.g., yoga programs, gym facilities with security guards). A participant explained that avoiding places that were unsafe was nuanced. “Avoidance can be bad but also good.”

#### Subtheme 4: Engagement and Empowerment

A few people (*N* = 3) said that engagement in programs that include the ability to volunteer and help others was an important way to challenge trauma. One said, “Helping someone less fortunate than you, it builds self-confidence.” Another person shared: “Well, I do a lot of outreach work, because of my past experience. I’ve had cancer twice, brain cancer. I get out in my community; I try to help people in my community.” A third participant said: “So I joined the consumer advisory, eventually I ran and became a board person. I started making changes in how my people were being treated. I also addressed how the staff treat people because you don’t know what people are coming in with.” These comments particularly highlighted the importance of grassroots efforts and community members coming together to address community trauma. Participating in these efforts seemed to empower them to make improvements in their lives.

## Discussion

In this study, we focused on the perceptions of African Americans with SMI in regards to trauma, diet, and exercise. Findings on the perceived influence of trauma on diet and exercise included how trauma contributed to emotional eating, interfered with appetite, resulted in hesitancy to exercise, contributed to people staying home rather than exercising, and furthered the use of substances. In particular, participants spoke about community violence and lack of safe spaces to engage in physical activities. In other research, perceptions of safety have been associated with reduced physical activity in neighborhoods (Brown et al., 2014), and increased stress (Burdette & Hill, 2008). Lack of neighborhood safety was a factor also identified by Siddiqi and colleagues (2011) in their systematic review of barriers to physical activity for African Americans; however, this study adds to the literature by highlighting additional ways that trauma is connected with both physical activity and diet-related behaviors. In contrast to Siddiqi and colleagues (2011), our participants also spoke about how mental health symptoms and substance use interfered with their ability to engage in wellness activities. Our participants described “hibernating” and having difficulty initiating healthy activities outside the home, which has not been addressed previously in the research literature. Overall, our study results confirmed the necessity of recognizing and understanding the influence of trauma on health and the accompanying need to integrate trauma-informed practices in human services.

Themes from this study also explored how diet and exercise programs can address trauma experienced by African Americans with SMI. Participants suggested that additional support, engaging in thoughtful/careful communication, teaching strategies to identify and avoid trauma, and providing opportunities for community engagement components that might help programming to be more trauma-informed and combat trauma. A recent review on trauma-informed physical activity programming (which was not specific to African Americans or individuals with mental illness) outlined ways that physical activity programs had incorporated trauma-informed practices in the design and delivery of their programming (Darroch et al., 2020). Similar to the participant perspectives from our study, communications from program staff were highlighted in the review as a trauma-informed component. Trauma-informed communications included use of calm demeanor, inviting language, emphasis on personal choice, and careful listening to participant preferences. Other programs noted the importance of ensuring safety by setting expectations, clarifying boundaries between participants and program staff, and having no-touch or low-touch policies (Darroch et al., 2020). Training of staff instructors, use of mindfulness exercises, and modifications to physical activity (e.g., option to keep eyes open during yoga pose) were other trauma-informed aspects described in these studies.

While these trauma-informed practices partially coincide with the results of the current study, several participants in our focus groups also discussed the importance of their own involvement in programming and the ability to help others as being important to address trauma. This suggests a preference for programs that are bidirectional and involve peer support components. While peer support interventions have historically focused on addressing mental health, the authors of a systematic review (Stubbs et al., 2016) located seven research studies on peer support-based physical health interventions for people with SMI. Overall, these studies were small and heterogenous, leading Stubbs and colleagues (2016) to conclude that more evidence is needed to establish the efficacy of peer support intervention for physical health.

### Implications

This study highlights the role of human services professionals in addressing the holistic needs of African Americans with SMI through interdisciplinary approaches. Given that African Americans with SMI are a disenfranchised population with high prevalence of both trauma exposure and health disparities, research and program development to address population-specific issues related to health and wellness is urgent. Previously developed wellness interventions for people with SMI have largely failed to produce results (Speyer et al., 2019). Human services professionals can develop diet and exercise interventions that explicitly identify trauma as a potential barrier, including an assessment of trauma and personalized plans to address trauma-related barriers. Training on trauma-sensitive communications and individualized program modifications for human service professionals (e.g., assignment to same-gendered staff) may increase the comfort level of program participants. Participants in our study said that emotional eating and loss of appetite were related to their trauma experiences. Human services professionals should recognize and explore these manifestations of trauma. Heightened concerns about community violence and the tendency to self-isolate due to mental health symptoms both suggest the need for human services that initially engage participants in their homes or provide improved access to engage people in initiating activities at safe locations outside their home. This might include developing virtual home exercise programs or providing transportation to exercise classes (e.g., see Lui et al., 2022 and Vincenzo et al., 2021).

Participants also recognized the importance of incorporating peer support into diet and exercise programs so they have the opportunity to learn from and help others. Peer supporters or paraprofessionals can complement the work of human services professionals by helping African Americans with SMI engage more fully with professional systems of care (e.g., see Stubbs et al., 2016). Human service professionals might also incorporate peer mentorships or buddy systems that enhance engagement and promote active bidirectional participation.

Our results highlight the importance of recognizing symptoms of trauma and other mental illness, and how they connect with health behaviors. As one research participant noted, it may be difficult to see the connection between trauma and diet. Human services programs can include psychoeducation on trauma in combination with referrals to, or integration with, mental health and substance use treatment. Researchers have stressed the crucial role of nutrition and exercise on people’s recovery from substance use disorders (Wiss, 2019). Human service professionals may collaborate with substance use treatment providers on intervention programs with a built-in component on addressing trauma and cultivating healthy lifestyle. Finally, previous research has shown that both physical activity and a nutritious diet can mitigate symptoms of mental illness (Firth et al., 2019; Rosenbaum et al., 2014, 2015). Human services that highlight the connection between physical and mental health and treat both aspects of health in a holistic way seem particularly promising.

### Limitations

This was a small qualitative study focusing on urban African Americans with SMI. Thus, results may not generalize or be representative of all individuals. There are many types of traumatic experiences; in this study, we did not conduct a systematic assessment of participants’ trauma or trauma-related diagnoses. Diagnosis of SMI, experience with trauma, and weight concerns were all self-reported and we collected limited information on participant demographics. Our decision to utilize focus groups likely also impacted the results; individual interviews might have allowed some individuals additional comfort in opening up on a sensitive topic, or allowed for more in-depth exploration of each topic. The methodology of this study precluded an examination of gender differences in the perceptions of trauma or other intersectional identities of research participants. Although we analyzed data in a systematic way, qualitative methods such as thematic analysis are subject to bias and qualitative data can be interpreted in various ways. Future research could seek to quantitatively validate our findings in a larger sample, or test trauma-informed programs for African Americans with SMI. Well-designed studies could identify key components that are most likely to engage these individuals in human services around diet and exercise. Our study was not grounded in a theoretical framework of trauma or engagement; thus, future research could attend to development or refinement of models to explain and predict the influence of trauma on diet and exercise programming.

### Conclusion

We hope this paper underscores the challenges that trauma may present for African Americans with SMI who are trying to improve diet and physical activity, as well as suggesting ways for human services programming to be more trauma-informed. Human service professionals who recognize and assess for trauma, and help their participants understand the connections and seek treatment for both mental and physical health can better meet the needs of this population. Due to symptoms and concerns about neighborhood safety, diet and exercise programming may need to provide individualized services to address unique challenges at the early stages of the engagement; provide modifications or alternatives to group classes outside the home; or problem-solve barriers to safe spaces. Further, mutual or professional peer support might help to better engage individuals in diet and exercise programming. To counteract the negative impact of social determinants of health, human services professionals should consider the need for advocacy within the profession to address issues of trauma and healthcare access for African Americans with SMI.

## References

[R1] Alegría M, Fortuna LR, Lin JY, Norris FH, Gao S, Takeuchi DT, Jackson JS, Shrout PE, & Valentine A (2013). Prevalence, risk, and correlates of posttraumatic stress disorder across ethnic and racial minority groups in the US. Medical Care, 51(12), 1114–1123. 10.1097/mlr.0000000000000007 24226308 PMC3922129

[R2] Allen J, Balfour R, Bell R, & Marmot M (2014). Social determinants of mental health. International Review of Psychiatry, 26(4), 392–407. 10.3109/09540261.2014.928270 25137105

[R3] Bartoli F, Crocamo C, Alamia A, Amidani F, Paggi E, Pini E, Clerici M, & Carrà G (2015). Posttraumatic stress disorder and risk of obesity: Systematic review and meta-analysis. Journal of Clinical Psychiatry, 76(10), e1253–e1261. 10.1097/hrp.0000000000000106 26528647

[R4] Bower KM, Thorpe RJ Jr., Rohde C, & Gaskin DJ (2014). The intersection of neighborhood racial segregation, poverty, and urbanicity and its impact on food store availability in the United States. Preventive Medicine, 58, 33–39. 10.1016/j.ypmed.2013.10.010 24161713 PMC3970577

[R5] Brown BB, Werner CM, Smith KR, Tribby CP, & Miller HJ (2014). Physical activity mediates the relationship between perceived crime safety and obesity. Preventive Medicine, 66, 140–144. 10.1016/j.ypmed.2014.06.021 24963894 PMC4134936

[R6] Burdette AM, & Hill TD (2008). An examination of processes linking perceived neighborhood disorder and obesity. Social Science & Medicine, 67(1), 38–46. 10.1016/j.socscimed.2008.03.029 18433964

[R7] Carliner H, Collins PY, Cabassa LJ, McNallen A, Joestl SS, & Lewis-Fernández R (2014). Prevalence of cardiovascular risk factors among racial and ethnic minorities with schizophrenia spectrum and bipolar disorders: a critical literature review. Comprehensive Psychiatry, 55(2), 233–247. 10.1016/j.comppsych.2013.09.009 24269193 PMC4164219

[R8] Cook JA, Razzano L, Jonikas JA, Swarbrick MA, Steigman PJ, Hamilton MM, Carter TM, & Santos AB (2016). Correlates of co-occurring diabetes and obesity among community mental health program members with serious mental illnesses. Psychiatric Services, 67(11), 1269–1271. 10.1176/appi.ps.201500219 27301761

[R9] Darroch FE, Roett C, Varcoe C, Oliffe JL, & Montaner GG (2020). Trauma-informed approaches to physical activity: A scoping study. Complementary Therapies in Clinical Practice, 41(3), 101224. 10.1016/j.ctcp.2020.101224 32819867

[R10] Fichtenberg C, Delva J, Minyard K, & Gottlieb LM (2020). Health and human services integration: generating sustained health and equity improvements. Health Affairs, 39(4), 567–573. 10.1377/hlthaff.2019.01594 32250685

[R11] Firth J, Marx W, Dash S, Carney R, Teasdale SB, Solmi M, Stubbs B, Schuch FB, Carvalho AF, Jacka F, & Sarris J (2019). The effects of dietary improvement on symptoms of depression and anxiety: A meta-analysis of randomized controlled trials. Psychosomatic Medicine, 81(3), 265–280. 10.1097/psy.0000000000000673 30720698 PMC6455094

[R12] Firth J, Rosenbaum S, Stubbs B, Gorczynski P, Yung AR, & Vancampfort D (2016). Motivating factors and barriers towards exercise in severe mental illness: a systematic review and meta-analysis. Psychological Medicine, 46(14), 2869–2881. 10.1017/s0033291716001732 27502153 PMC5080671

[R13] Frazer E, Mitchell RA Jr., Nesbitt LS, Williams M, Mitchell EP, Williams RA, & Browne D (2018). The violence epidemic in the African American community: a call by the National Medical Association for comprehensive reform. Journal of the National Medical Association, 110(1), 4–15. 10.1016/j.jnma.2017.08.009 29510842

[R14] Hampton-Anderson JN, Carter S, Fani N, Gillespie CF, Henry TL, Holmes E, Lamis DA, LoParo D, Maples-Keller JL, Powers A, Sonu S, & Kaslow NJ (2021). Adverse childhood experiences in African Americans: Framework, practice, and policy. American Psychologist, 76(2), 314–325. 10.1037/amp0000767 33734797

[R15] Janssen EM, McGinty EE, Azrin ST, Juliano-Bult D, & Daumit GL (2015). Review of the evidence: prevalence of medical conditions in the United States population with serious mental illness. General Hospital Psychiatry, 37(3), 199–222. 10.1016/j.genhosppsych.2015.03.004 25881768 PMC4663043

[R16] Kubzansky LD, Bordelois P, Jun HJ, Roberts AL, Cerda M, Bluestone N, & Koenen KC (2014). The weight of traumatic stress: a prospective study of posttraumatic stress disorder symptoms and weight status in women. JAMA Psychiatry, 71(1), 44–51. 10.1001/jamapsychiatry.2013.2798 24258147 PMC4091890

[R17] Lane JM, & Davis BA (2022). Food, physical activity, and health deserts in Alabama: the spatial link between healthy eating, exercise, and socioeconomic factors. GeoJournal, 87(6), 5229–5249. 10.1007/s10708-021-10568-2

[R18] Marshall C, & Rossman G (1999). Designing qualitative research (3rd ed.). Sage Publications.

[R19] Mihelicova M, Brown M, & Shuman V (2018). Trauma-informed care for individuals with serious mental illness: an avenue for community psychology’s involvement in community mental health. American Journal of Community Psychology, 61(1–2), 141–152. 10.1002/ajcp.12217 29266247

[R20] Miller ML, Strassnig MT, Bromet E, Depp CA, Jonas K, Lin W, Moore RC, Patterson TL, Penn DL, Pinkham AE, Kotov RA, & Harvey PD (2021). Performance-based assessment of social skills in a large sample of participants with schizophrenia, bipolar disorder and healthy controls: Correlates of social competence and social appropriateness. Schizophrenia Research, 236, 80–86. 10.1016/j.schres.2021.08.012 34425381 PMC10857848

[R21] Mueser KT, Salyers MP, Rosenberg SD, Goodman LA, Essock SM, Osher FC, Swartz MS, Butterfield MI, & 5 Site Health and Risk Study Research Committee. (2004). Interpersonal trauma and posttraumatic stress disorder in patients with severe mental illness: Demographic, clinical, and health correlates. Schizophrenia Bulletin, 30(1), 45–57. 10.1093/oxfordjournals.schbul.a007067 15176761

[R22] Myers HF, Wyatt GE, Ullman JB, Loeb TB, Chin D, Prause N, Zhang M, Williams JK, Slavich GM, & Liu H (2015). Cumulative burden of lifetime adversities: Trauma and mental health in low-SES African Americans and Latino/as. Psychological Trauma: Theory, Research, Practice, and Policy, 7(3), 243–251. 10.1037/a0039077 25961869 PMC4445692

[R23] National Center for Health Statistics. (2019). Health, United States, 2018. 31944640

[R24] Robins LB, Johnson KF, Duyile B, Gantt-Howrey A, Dockery N, Robins SD, & Wheeler N (2022). Family counselors addressing social determinants of mental health in underserved communities. The Family Journal, 31(2), 213–221. 10.1177/10664807221132799

[R25] Rocks T, Teasdale SB, Fehily C, Young C, Howland G, Kelly B, & O’Neil A (2022). Effectiveness of nutrition and dietary interventions for people with serious mental illness: systematic review and meta-analysis. Medical Journal of Australia, 217, S7–S21. 10.5694/mja2.51680 PMC9828433 36183316

[R26] Rosenbaum S, Tiedemann A, Sherrington C, Curtis J, & Ward PB (2014). Physical activity interventions for people with mental illness: a systematic review and meta-analysis. Journal of Clinical Psychiatry, 75(9), 964–974. 10.4088/jcp.13r08765 24813261

[R27] Rosenbaum S, Vancampfort D, Steel Z, Newby J, Ward PB, & Stubbs B (2015). Physical activity in the treatment of post-traumatic stress disorder: a systematic review and meta-analysis. Psychiatry Research, 230(2), 130–136. 10.1016/j.psychres.2015.10.017 26500072

[R28] Sayer J, Paniagua D, Ballentine S, Sheehan L, Carson M, Nieweglowski K, Corrigan P, & Community-Based Participatory Research (CBPR) Team. (2019). Perspectives on diet and physical activity among urban African Americans with serious mental illness. Social Work in Health Care, 58(5), 509–525. 10.2337/diacare.26.9.2643 30907271 PMC6658098

[R29] Sheehan L, Ballentine S, Washington L, Canser M, Connor J, Jones R, Laster E, Muhammad K, Noble S, Smith R, Walley G, Kundert C, & Corrigan PW (2021). Implementing community-based participatory research among African Americans with serious and persistent mental illness: A qualitative study. Gateways: International Journal of Community Research and Engagement, 14(1), 1–19. 10.5130/ijcre.v14i1.6894

[R30] Siddiqi Z, Tiro JA, & Shuval K (2011). Understanding impediments and enablers to physical activity among African American adults: a systematic review of qualitative studies. Health Education Research, 26(6), 1010–1024. 10.1093/her/cyr068 21873458

[R31] Speyer H, Jakobsen AS, Westergaard C, Nørgaard HCB, Pisinger C, Krogh J, Hjorthøj C, Nordentoft M, Gluud C, Correll CU, & Jørgensen KB (2019). Lifestyle interventions for weight management in people with serious mental illness: a systematic review with meta-analysis, trial sequential analysis, and meta-regression analysis exploring the mediators and moderators of treatment effects. Psychotherapy and Psychosomatics, 88(6), 350–362. 10.1159/000502293 31522170

[R32] Stolzenberg L, D’Alessio SJ, & Flexon JL (2019). The impact of violent crime on obesity. Social Sciences, 8(12), 329. 10.3390/socsci8120329

[R33] Stubbs B, Williams J, Shannon J, Gaughran F, & Craig T (2016). Peer support interventions seeking to improve physical health and lifestyle behaviours among people with serious mental illness: A systematic review. International Journal of Mental Health Nursing, 25(6), 484–495. 10.1111/inm.12256 27600483

[R34] U.S. Department of Health and Human Services. (2014). SAMHSA’s concept of trauma and guidance for a trauma-informed approach (pp. 1–20). Rebuilding the Launchpad: Serving Students During Covid Resource Library.

[R35] Vincenzo JL, Hergott C, Schrodt L, Rohrer B, Brach J, Tripken J, Shirley KD, Sidelinker JC, & Shubert TE (2021). Capitalizing on virtual delivery of community programs to support health and well-being of older adults. Physical Therapy, 101(4). 10.1093/ptj/pzab001 PMC8023634 33439254

[R36] Wiss DA (2019). Chapter 2 - The role of nutrition in addiction recovery: What we know and what we don’t. In Danovitch I & Mooney LJ (Eds.), The Assessment and Treatment of Addiction (pp. 21–42). Elsevier. 10.1016/b978-0-323-54856-4.00002-x

[R37] Wright AW, Austin M, Booth C, & Kliewer W (2017). Systematic review: exposure to community violence and physical health outcomes in youth. Journal of Pediatric Psychology, 42(4), 364–378. 10.1093/jpepsy/jsw088 27794530

[R38] Yau YHC, & Potenza MN (2013). Stress and eating behaviors. Minerva Endocrinologica, 38(3), 255–267. 24126546 PMC4214609

